# Prioritizing Congenital Syphilis Control in South China: A Decision Analytic Model to Inform Policy Implementation

**DOI:** 10.1371/journal.pmed.1001375

**Published:** 2013-01-22

**Authors:** Nicholas X. Tan, Chara Rydzak, Li-Gang Yang, Peter Vickerman, Bin Yang, Rosanna W. Peeling, Sarah Hawkes, Xiang-Sheng Chen, Joseph D. Tucker

**Affiliations:** 1 Harvard Institute for Global Health, Cambridge, Massachusetts, United States of America; 2 Department of Radiology, Perelman School of Medicine at the University of Pennsylvania, Philadelphia, Pennsylvania, United States of America; 3 Guangdong Provincial Center for STI Prevention and Control, Guangzhou, China; 4 London School of Hygiene and Tropical Medicine, London, United Kingdom; 5 University College London, London, United Kingdom; 6 National STD Control Center, Nanjing, China; 7 UNC Project—China, Guangzhou, China; Harvard School of Public Health, United States of America

## Abstract

**Background:**

Syphilis is a major public health problem in many regions of China, with increases in congenital syphilis (CS) cases causing concern. The Chinese Ministry of Health recently announced a comprehensive 10-y national syphilis control plan focusing on averting CS. The decision analytic model presented here quantifies the impact of the planned strategies to determine whether they are likely to meet the goals laid out in the control plan.

**Methods and Findings:**

Our model incorporated data on age-stratified fertility, female adult syphilis cases, and empirical syphilis transmission rates to estimate the number of CS cases associated with prenatal syphilis infection on a yearly basis. Guangdong Province was the focus of this analysis because of the availability of high-quality demographic and public health data. Each model outcome was simulated 1,000 times to incorporate uncertainty in model inputs. The model was validated using data from a CS intervention program among 477,656 women in China. Sensitivity analyses were performed to identify which variables are likely to be most influential in achieving Chinese and international policy goals. Increasing prenatal screening coverage was the single most effective strategy for reducing CS cases. An incremental increase in prenatal screening from the base case of 57% coverage to 95% coverage was associated with 106 (95% CI: 101, 111) CS cases averted per 100,000 live births (58% decrease). The policy strategies laid out in the national plan led to an outcome that fell short of the target, while a four-pronged comprehensive syphilis control strategy consisting of increased prenatal screening coverage, increased treatment completion, earlier prenatal screening, and improved syphilis test characteristics was associated with 157 (95% CI: 154, 160) CS cases averted per 100,000 live births (85% decrease).

**Conclusions:**

The Chinese national plan provides a strong foundation for syphilis control, but more comprehensive measures that include earlier and more extensive screening are necessary for reaching policy goals.

*Please see later in the article for the Editors' Summary*

## Abstract 2

Nicholas Tan and colleagues use a decision analytic model to quantify the impact of the ten-year national syphilis control plan in China and conclude that earlier and more extensive screening are also necessary for reaching policy goals.

## Editors' Summary

**Background:**

Every year, 1.5 million pregnant women are infected with syphilis, a bacterial infection that is usually transmitted during sexual contact but that can also pass from a mother to her unborn child. In many of these women, the disease is detected through routine antenatal testing and is successfully treated with penicillin. But among those women who are not treated, about half experience adverse outcomes—the death of their baby during early or late pregnancy (fetal death and stillbirth, respectively) or soon after birth (neonatal death), or the birth of an infected baby. Babies born with syphilis (congenital syphilis) often fail to thrive and can develop problems such as blindness, deafness, and seizures if not treated. In 2008, syphilis in pregnancy contributed to 305,000 fetal deaths, stillbirths, and neonatal deaths, and 215,000 babies were affected by other adverse consequences of congenital syphilis. Yet congenital syphilis is simple and cheap to eliminate—a few injections of penicillin can clear the infection in a pregnant woman, and screening all pregnant women for syphilis is feasible even in low-resource settings.

**Why Was This Study Done?:**

Congenital syphilis has recently reemerged in China. In the 1990s, there were very few cases of congenital syphilis in China, but by 2009, the reported incidence of congenital syphilis had risen to 139 cases per 100,000 live births. In 2010 the Chinese Ministry of Health announced a ten-year National Syphilis Prevention and Control Plan (NSCP) that, in line with World Health Organization (WHO) recommendations, aims to reduce the incidence of congenital syphilis to fewer than 30 cases per 100,000 live births by 2015 and to fewer than 15 cases per 100,000 live births by 2020. China's strategy for achieving these targets includes increasing prenatal syphilis screening coverage to 80% in urban areas and 60% in rural areas, increasing treatment rates among infected women to 90% in urban areas and 70% in rural areas, and increasing syphilis awareness among adults. But will this strategy be sufficient? Here, the researchers develop a mathematical model to quantify the likely impact of the NSCP's strategy on the incidence of congenital syphilis in southern China.

**What Did the Researchers Do and Find?:**

The researchers developed a decision analytic model in which women move through a sequence of health states from uninfected, through infection and pregnancy, and to the development of congenital syphilis, and fed data collected in Guangdong Province between 2005 and 2008 on women's fertility, female syphilis cases, and syphilis transmission rates into the model. The researchers checked that their model provided estimates of the incidence of congenital syphilis that matched the reported incidence for Guangdong Province (internal validation) and the reported incidence in a congenital syphilis intervention program in Shenzhen, Guangdong (external validation). They then used their model to identify which parts of the NSCP strategy are likely to have the greatest effect on the incidence of congenital syphilis. Increasing prenatal screening coverage was the single most effective strategy for the reduction of congenital syphilis, but neither this strategy alone nor implementation of the whole NPSC strategy achieved the plan's target outcomes. By contrast, a four-pronged approach (95% coverage of prenatal screening, 75% of screening during the first two-thirds of pregnancy, 95% treatment completion, and having an accurate screening test) reduced the estimated incidence of congenital syphilis cases to 27 per 100,000 live births.

**What Do These Findings Mean?:**

These findings suggest that although the NSCP is a strong foundation for control of congenital syphilis in China, more comprehensive measures will be needed to reach the plan's policy goals. In particular, the findings suggest that earlier and more extensive screening will be needed to reduce the incidence of congenital syphilis to below 30 cases per 100,000 live births, WHO's benchmark for congenital syphilis control. The accuracy of these findings is limited by the assumptions included in the model and by the data fed into it. Moreover, because the data included in the model came from Guangdong Province, these findings may not apply elsewhere in China or in other countries. Nevertheless, this study illustrates the importance of using multi-pronged approaches to address the complex problem of congenital syphilis control and identifies some strategies that are likely to improve the control of this important public health problem.

**Additional Information:**

Please access these websites via the online version of this summary at http://dx.doi.org/10.1371/journal.pmed.1001375.

- The World Health Organization provides information on sexually transmitted infections, including details of its strategy for the global elimination of congenital syphilis, the investment case for the elimination of mother-to-child transmission of syphilis, and regional updates on progress towards elimination (some information is available in several languages)
- The US Centers for Disease Control and Prevention has a fact sheet on syphilis
- The UK National Health Service Choices website also has a page on syphilis
- MedlinePlus provides information on congenital syphilis and links to additional syphilis resources (in English and Spanish)
- Haiti: Congenital Syphilis on the Way Out is a YouTube video describing the introduction of rapid diagnostic tests for syphilis in remote parts of Haiti

## Introduction

Congenital syphilis (CS) has reemerged in China as a common, preventable cause of stillbirth, neonatal death, low birth weight, and irreversible congenital malformations [1]–[4]. In response to the resurgent syphilis epidemic, the Chinese Ministry of Health recently announced a comprehensive 10-y National Syphilis Prevention and Control Plan (NSCP). Averting CS cases stands at the center of this mandate, with explicit strategies that focus on improving prenatal syphilis screening coverage (target of 80% coverage in urban areas and 60% coverage in rural areas), increasing treatment rates in infected pregnant woman (target of 90% coverage in urban areas and 70% coverage in rural areas), and increasing syphilis awareness among adults [5]. The NSCP aims to reduce the number of newly reported CS cases to less than 30 cases per 100,000 live births by 2015 and to less than 15 cases per 100,000 live births by 2020. In 2009, there were 139 reported CS cases per 100,000 live births. The 2015 goal represents a 78.4% reduction from the 2009 reported caseload. The 2015 goal is consistent with recommendations from a working group led by the World Health Organization (WHO) and critical to achieving the 2020 objective of fewer than 15 CS cases per 100,000 live births [6]. Prioritizing policy strategies and understanding the key drivers of adverse outcomes associated with syphilis in pregnancy are essential for meeting these policy goals.

The lack of population-based data on adverse outcomes associated with syphilis infection during pregnancy in China has generated ambiguity about the extent of this public health problem and the organization of optimal control strategies. Although syphilis is associated with other adverse outcomes (neonatal death, premature birth, and low birth weight), this study focused on measuring CS cases because of the availability of robust data and a direct connection to NSCP goals [1]–[4]. Decision analytic methods provide a quantitative foundation for understanding the scope of CS in China and have been widely used to explore syphilis burden in other contexts [7],[8]. Drawing on empirical Chinese studies, adult syphilis case rates, and age-stratified fertility data, we estimate the reduction in CS burden among pregnant women with syphilis in Guangdong Province, China, with the expectation of achieving NSCP goals.

## Methods

### Analytic Overview

**Figure 1:**
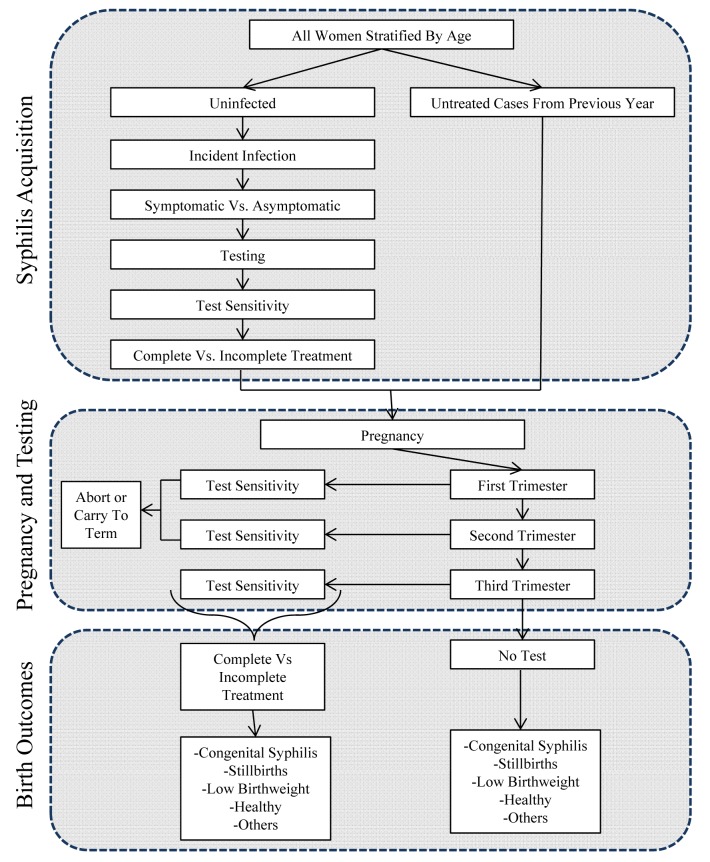
Decision analytic model. The figure illustrates the series of transition states leading to various adverse birth outcomes attributable to CS.

Based on published literature and Guangdong surveillance data, a decision analytical tree approach was used to model the acquisition and natural history of syphilis from infection to pregnancy to congenital birth outcomes. The Chinese case definition of CS was used, requiring positive treponemal and non-treponemal serologies in addition to a consistent clinical history. The decision analytical model consists of sequences of possible health states that simulate the pathways leading to CS within a population (Figure 1). Each health transition state is separated from other states by a transition state probability (Table 1). The decision analytical model consists of four main health events: syphilis acquisition, pregnancy, prenatal screening, and birth outcome.

**Table 1:** Transition state probabilities with uncertainty.

Variable	Base Case	Range	Model Uncertainty Range	Sources
**Primary and secondary syphilis**	Age-dependent			
Incidence rate		15–19 y old: 0.015%–0.017%	±5%	Guangdong Provincial Center for STI Prevention and Control [11]
		20–24 y old: 0.068%–0.083%		
		25–29 y old: 0.104%–0.114%		
		30–34 y old: 0.064%–0.077%		
		35–39 y old: 0.038%–0.052%		
		40–45 y old: 0.030%–0.052%		
		46–50 y old: 0.022%–0.034%		
Prevalence rate		0.47%–0.83%		
**Test-seeking behavior** a	20%	—	±20%	[16],[48]
**Non-treponemal test sensitivity**	95%			
Primary syphilis		77%–86%	±5%	[15]
Secondary syphilis		100%		
Latent syphilis		95%–100%		
**Non-treponemal test specificity**	98%	98%	±5%	[15]
**Adult syphilis treatment completion rate** b	80%	—	±5%	[36]
**Pregnant women treatment completion rate** b	85%	—	±5%	[36]
**Birth rate** c	Age Dependant	0.06%–11%	—	2000 and 2005 China census
**Total prenatal testing coverage**	57%	—	±5%	Guangdong Provincial Syphilis Test Capacity Project
**Prenatal testing across trimesters**				
1st trimester	23%	17%–25%	±5%	[25],[49]
2nd trimester	23%	17%–25%		
3rd trimester	28%	20%–40%		
**Abortion rate for positive prenatal syphilis tests**	13.6%	13.6%–16.9%	±20%	[8],[10]
**Complete treatment: CS outcomes**				
Treatment in 1st trimester	0.03%	0.03%–9%	±10%	[4],[13],[36],[50]–[52]
Treatment in 2nd trimester	5%	5%–30%		
Treatment in 3rd trimester	30%	28%–60%		
**No treatment: CS outcomes**	30%	30%–77%	±10%	[8],[10],[36],[51],[52]

a Approximately 50% of patients who are infected with primary syphilis report symptoms [16]. A population-representative survey of sexual health in China found that among all those with STI symptoms, approximately 40% actively seek testing [48].

b Complete treatment is defined as the completion of three doses of penicillin G benzathine or its equivalent [53].

c Average birth rates from the 2000 and 2005 China censuses were used.

The cycle length for the model simulation was 1 y. Within each year, the female population was stratified into seven age groups: 15–19 y old, 20–24 y old, 25–29 y old, 30–34 y old, 35–39 y old, 40–44 y old, and 45–49 y old. The total estimated CS burden was the sum of infants with CS born to pregnant women across all seven age groups.

The model was validated both internally and externally according to International Society for Pharmacoeconomics and Outcomes Research modeling guidelines [9], using, respectively, Guangdong syphilis surveillance data from years 2005 to 2008 and empirical data from a CS intervention program in Guangdong Province that screened 477,656 pregnant women citywide from 2002 to 2005 [10]. For both validation exercises, the set of the data used for validation purposes was separate from data used for later input parameter estimates.

### Model

The decision analytic model was built and programmed using TreeAge Pro 2011 (TreeAge Software). Women entered the model through one of two possible starting states—uninfected or infected. The proportion of women in each starting state was determined by syphilis prevalence, represented by provincial data on syphilis burden per 100,000 people.

For uninfected women, the probability of acquiring syphilis was represented by the primary and secondary syphilis incidence rates. All sexually transmitted infection (STI) data for syphilis were obtained from the province-wide mandatory reporting system organized by the Guangdong Provincial Center for STI Prevention and Control [11].

### Data

Table 1 lists the transition state probabilities used in the decision analytic model and respective data ranges. The base case was based on disease and demographic data from 2009. Conservative parameter estimates were used for base case values but were varied across the upper and lower ranges of reported values and values from sensitivity analyses to ensure that the results were robust. Syphilis prevalence rates refer to primary and secondary syphilis cases. Latent and tertiary syphilis cases were excluded because mother–child transmission rates are very low for these stages. Treatment completion was defined as three doses of penicillin G benzathine or its equivalent. Whenever possible, we used data from randomized controlled trials and epidemiologic studies specific to the population under evaluation. In addition, model inputs were drawn from Chinese studies. We drew from a large body of literature pertaining to the natural history and epidemiology of syphilis in pregnancy, using data from large and high-quality studies. Incidence and prevalence rates utilized as model inputs were predominantly from urban clinics, where there is a greater burden of syphilis and better infrastructure to respond to diagnosed cases. In instances where data specific to the analysis population were limited, relevant applicable data from the literature for populations that most closely approximated our study population characteristics, while providing the best available parameter estimate, were used.

#### Sensitivity analyses

We performed 1,000 simulations using parameter values randomly sampled from uniform distributions between the upper and lower ranges of all health state variables to ensure that the model was stable and robust (ranges used are shown in Table 1). For model parameters obtained from strong, geographically relevant, empirical sources (e.g., syphilis incidence rates, test sensitivity and specificity, treatment completion, testing coverage, and time of testing), a ±5% uncertainty interval was applied. For the CS outcome probability, a ±10% uncertainty interval was applied because there was some variation from study to study, although all studies considered were Chinese studies. For behavioral measures that lacked reliable and accurate data (e.g., test-seeking behavior and abortion rate), a ±20% uncertainty interval was applied. For each individual policy strategy (see “Strategies” below), the percent reduction was computed; the point estimate was the mean of these percent reduction estimates. The confidence interval was taken from the 2.5th and 97.5th percentiles. Policy strategy, CS outcome probability, and prevalence rate were the only health state variables that resulted in more than a 4% change in the number CS cases.

In addition, sensitivity analyses were performed on all variables that corresponded to each policy strategy. The individual policy strategies that were associated with the largest number of CS cases averted were identified. Multivariate sensitivity analyses were then performed to identify the combinations of individual strategies associated with the greatest decreases in CS cases.

#### Validation exercises

Two exercises were used to validate the model, involving 2005–2008 CS incidence in Guangdong Province and a CS intervention program in Shenzhen City.

### Internal Validation: Historical Guangdong Province Reported CS Cases

After model testing and debugging, an internal validation was performed with data for a 4-y period (2005–2008) from the Guangdong Provincial Center for STI Prevention and Control. This constitutes an internal validation because data exist on both model inputs (demographic data and adult syphilis incidence and prevalence rates) and model outputs (incident annual CS cases) over the same time period [9]. This time period was chosen because there were major administrative changes in reporting from 2003 to 2004 [12]. Demographic data, adult syphilis incidence rates, and adult syphilis prevalence rates for each respective year were used. Demographic data consisted of birth rates and female population sizes for each age-stratified group obtained from the 2000 and 2005 China censuses. Adult syphilis incidence and adult syphilis prevalence rates were obtained from the Guangdong Provincial Center for STI Prevention and Control [11]. The number of reported CS cases was divided by the number of estimated CS cases to calculate reporting rates. The validation exercise showed similar trends for model estimates and reported CS cases across all 4 y, with reporting rates of 54%, 69%, 78%, and 82% for 2005, 2006, 2007, and 2008, respectively. Reporting rates from 2005 to 2008 were stable, with an average reporting rate of 71% (95% CI: 59%, 83%).

### External Validation: Shenzhen CS Intervention Program

The model was validated against published data from a CS intervention among 477,656 pregnant women in Shenzhen. This external validation tested the model outcomes against a pilot study conducted in a Guangdong city. Model inputs were obtained from Cheng et al. [10] and did not include inputs used to make base case model assumptions. From 2002 to 2005, the Shenzhen government initiated a citywide syphilis prevention program for pregnant women to curb the sharp rise in CS cases [10]. The program provided free syphilis screening, treatment, and follow-up for all pregnant women. Demographic data, prenatal screening coverage, syphilis prevalence and incidence rates, and treatment completion rates that were specific to Shenzhen were inputted into the decision analytic model [8],[10],[13],[14]. Data used for validation of the model consisted of a subset of the original data that was not used for producing policy parameter estimates. Shenzhen demographic data were obtained from the 2000 and 2005 China censuses. Shenzhen adult syphilis incidence rates were obtained from the Guangdong Provincial Center for STI Prevention and Control. The adult syphilis prevalence in Shenzhen was obtained from a published study that investigated syphilis in many populations [14]. The intervention program increased prenatal screening coverage to 94% and the treatment completion rate to 92%. Based on all of the above inputs, the model estimated 29 (95% CI: 28, 30) CS cases per 100,000 live births over the course of the entire Guangdong CS intervention program, compared to the 27 (95% CI: 24, 30) cases per 100,000 reported in the empirical data, after accounting for loss to follow-up. This represents a difference of 7% between the model and the empirical Shenzhen data.

### Strategies

The NSCP approach focuses on four main strategies [5]: running educational campaigns aimed at educating the public on safer sex practices to decrease syphilis incidence rates, increasing prenatal screening coverage, increasing treatment completion rates in rural and urban places, and improving the medical knowledge and skills of health professionals. In addition to these four strategies, we investigated three strategies that have been successful in other countries and settings [4]: increasing the percentage of pregnant women who receive syphilis testing early, i.e., in the first and second trimester; introducing new testing technologies that might improve test sensitivity and specificity; and running educational campaigns aimed at reducing the social stigma associated with STIs and promoting recognition of syphilis symptoms among the general adult population, to increase test-seeking rates.

The number of CS cases expected with the isolated implementation of each policy strategy was estimated. Single policy strategies were combined into combination strategies, and all possible combinations were explored. Analyzing the CS case reductions from combined strategies is crucial because the overall outcome from combination strategies depends on the interaction between individual strategies. The reduction in the number of CS cases that would result from the successful implementation of the NSCP was computed and compared against NSCP and WHO targets, and against the combination policies that resulted in the highest CS case reductions. This analysis enables the advocacy of effective strategies outside of the scope of the NSCP.

## Results

### Base Case Results

The base case year 2009 had a model estimate of 2,079 new CS cases, corresponding to 184 (95% CI: 172, 196) CS cases per 100,000 live births. Given the 1,567 CS cases reported by the Guangdong Provincial Center for STI Prevention and Control surveillance system, this represents an estimated reporting rate of 75.5% if the model estimate is correct. Table 1 includes all base case model inputs used in this analysis. Conservative inputs within the respective input ranges were used to avoid the possibility of overestimating CS cases.

### One-Way Sensitivity Analyses: Single CS Policy Strategies

#### Total prenatal screening coverage

Increasing prenatal screening coverage to 95% was the most influential individual strategy in reducing CS burden (Table 2). In 2009, the estimated coverage for screening of pregnant women was 57%, with an estimated 184 (95% CI: 172, 196) CS cases per 100,000 live births. Increasing prenatal screening coverage to 95% while leaving all other aspects of the 2009 model inputs constant resulted in an estimated 78 (95% CI: 73, 83) CS cases per 100,000 live births. This policy strategy alone averted 58% (95% CI: 55, 60) of CS cases compared to the base case. Figure S1 shows the linear relationship between increasing prenatal screening coverage and total CS cases over a range of values.

**Table 2:** Summary of estimated outcomes with parameters from the base case scenario, single CS policy strategies, and combination strategies.

Factor/Outcome	2009 Base Case	Single CS Policy Strategies						Most Effective Combination CS Policy Strategies			
		Increased Prenatal Screening Coverage	Earlier Prenatal Testing	Decreased Syphilis Incidence	Increased Treatment Completion	Improved Test Sensitivity and Specificity	Increased Test Seeking	Two-Pronged Screening Strategy	Three-Pronged Strategy 1	Three-Pronged Strategy 2	Four-Pronged Strategy
**Base case**	Refer to Table 1	Total prenatal coverage: 57%	1st trimester testing: 23%; 2nd trimester testing: 23%; 3rd trimester testing: 28%	Age-specific	Adult: 80%; pregnant women: 85%	Test sensitivity: 95%; test specificity: 98%	40%				
**Parameter change based on policy**	—	Total prenatal coverage: 95%	1st trimester testing: 30%; 2nd trimester testing: 30%; 3rd trimester testing: 10%	Uniform 50% decrease	Adult: 95%; pregnant women: 95%	Test sensitivity: 99%; test specificity: 99%	80%	(1) Increased prenatal screening coverage; (2) earlier prenatal testing	(1) Increased prenatal screening coverage; (2) earlier prenatal testing; (3) decreased syphilis incidence	(1) Increased prenatal screening coverage; (2) earlier prenatal testing; (3) increased treatment completion	(1) Increased prenatal screening coverage; (2) earlier prenatal testing; (3) increased treatment completion; (4) improved test sensitivity and specificity
**Feasibility**	—	Demonstrated globally including China [4],[10],[22]	Demonstrated in low-income settings [36]	None	Demonstrated globally including China [4],[10],[22]	None	None				
**CS model outcomes (cases per 100,000 live births)**	Model outcome: 184; 95% CI: 172, 196; reported: 139	78; 95% CI: 73, 83	160; 95% CI: 149, 171	172; 95% CI: 160, 184	175; 95% CI: 163, 187	176; 95% CI: 164, 188	176; 95% CI: 165, 187	50; 95% CI: 46, 54	50; 95% CI: 46, 54	35; 95% CI: 32, 38	27; 95% CI: 24, 30
**Reduction** a **(from base case)**	—	58%; 95% CI: 55, 60	13%; 95% CI: 7, 19	7%; 95% CI: 0, 13	5%; 95% CI: −2, 11	4%; 95% CI: −2, 11	4%; 95% CI: −2, 11	73%; 95% CI: 71, 75	73%; 95% CI: 71, 75	81%; 95% CI: 79, 83	85%; 95% CI: 84, 87

a These values were compared to the 2009 base case estimate.

#### Earlier prenatal screening

The second most influential individual strategy was earlier prenatal screening. The proportion of women screened during their first trimester and second trimester was increased from 23% to 30% while holding total prenatal coverage constant at the base case value of 57%. Third trimester screening was reallocated to the first and second trimesters to keep the total prenatal screening coverage constant. Hence, this measure isolates the effect of reaching out to already accessible women earlier in their pregnancy without increasing testing coverage or affecting other variables. This strategy was associated with 160 (95% CI: 149, 171) CS cases per 100,000 births and a 13% (95% CI: 7, 19) reduction compared to the base case (Table 2).

#### Syphilis incidence

The third most influential individual strategy was decreasing adult syphilis incidence, a strategy in which the NSCP has substantially invested. Syphilis incidence was decreased by 100% to 0% in the model, and it was found that after the 50% point, CS case reductions were negligible. Hence a 50% decrease was used as the maximum policy parameter target. This strategy was associated with 172 (95% CI: 160, 184) CS cases per 100,000 births and a 7% (95% CI: 0, 13) reduction compared to the base case (Table 2).

#### Treatment completion

The fourth most influential individual strategy was increasing treatment completion rates. Treatment completion rate is defined as the portion of pregnant women with syphilis overall who receive three doses of penicillin G benzathine or its equivalent. The treatment completion rate for pregnant women was increased in the model from 85% to 95%, while the treatment completion rate for all non-pregnant adults was increased from 80% to 95%. This strategy was associated with 175 (95% CI: 163, 187) CS cases per 100,000 births and a 5% (95% CI: −2, 11) reduction compared to the base case (Table 2).

#### Test sensitivity and specificity

The fifth most influential individual strategy was improving test sensitivity and specificity. Current non-treponemal test sensitivity and specificity are 95% and 98%, respectively [15]. This policy parameter aims to increase both test sensitivity and specificity to 99%, which is the upper limit demonstrated in US Centers for Disease Control and Prevention studies [15]. This strategy was associated with 176 (95% CI: 164, 188) CS cases per 100,000 births and a 4% (95% CI: −2, 11) reduction compared to the base case (Table 2).

#### Test-seeking behavior among adult population

The least influential policy strategy was increasing test-seeking behavior. Test-seeking behavior is limited by biological symptoms, with only 50% of patients infected with primary and secondary syphilis reporting symptoms [16]. Test-seeking behavior was increased from 40% to 100%. When test seeking reached 80%, it resulted in the greatest CS case reductions, but above that point, increases in test seeking resulted in negligible CS case reductions.

### Multi-Way Sensitivity Analyses: Combination CS Policy Strategies

We performed multi-way sensitivity analyses to identify the combinations of strategies that averted the most CS cases for multi-pronged policy strategies. Feasibility was also taken into account when developing combination CS policy strategies. All combinations of two-pronged, three-pronged, four-pronged, and five-pronged strategies were tested. The most effective combinations are reported (Table 2). No five-pronged policy strategy reduced more CS cases than the most effective four-pronged combination.

#### Two-pronged strategy

**Figure 2:**
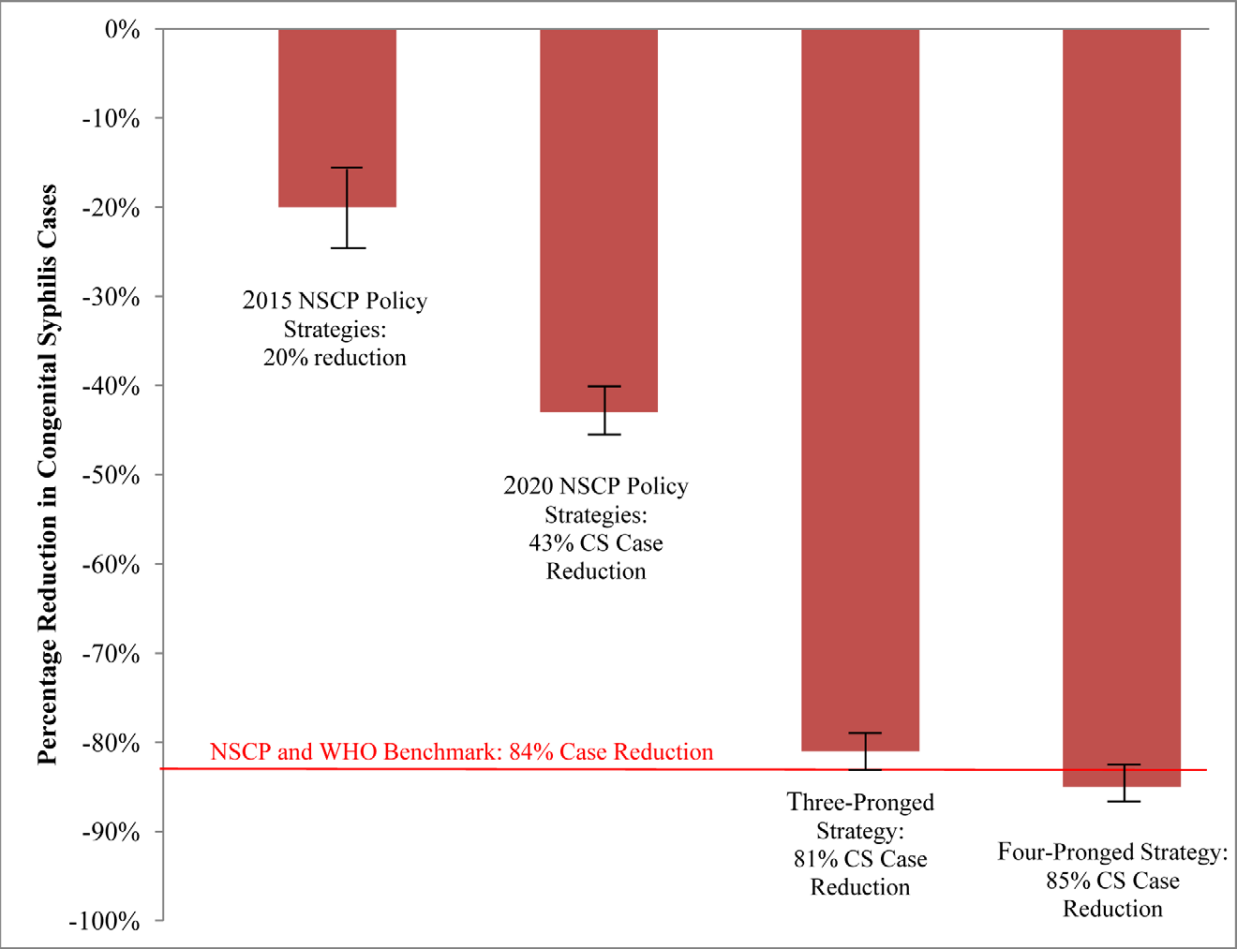
National syphilis control plan and combination strategy estimated outcomes with 95% CIs. The three-pronged strategy consists of increased prenatal screening coverage, earlier prenatal testing, and increased treatment completion rate. The four-pronged strategy consists of increased prenatal screening coverage, earlier prenatal testing, increased treatment completion rate, and increased test sensitivity and specificity. These values were compared to the 2009 base case estimate.

The two-pronged strategy that averted the most CS cases consisted of increased prenatal screening coverage and earlier prenatal testing, which were also the two most influential individual strategies. Prenatal screening coverage was increased from 57% to 95% and prenatal screening was performed 75% of the time in the first and second trimester. This screening strategy was associated with 50 (95% CI: 46, 54) CS cases per 100,000 births and a 73% (95% CI: 71, 75) reduction compared to the base case (Table 2; Figure 2).

#### Three-pronged strategy

The three-pronged strategy that averted the most CS cases consisted of increased prenatal screening coverage, earlier prenatal testing, and increased treatment completion. Total prenatal screening coverage was increased from 57% to 95%, prenatal screening was performed 75% of the time in the first and second trimester, and treatment completion rates were 95%. This three-pronged strategy was associated with 35 (95% CI: 32, 38) CS cases per 100,000 births and an 81% (95% CI: 79, 83) reduction compared to the base case (Table 2; Figure 2).

The three-pronged strategy that averted the second most CS cases consisted of increased prenatal screening coverage, earlier prenatal testing, and decreased adult syphilis incidence. Prenatal screening coverage metrics were similar to the above, and syphilis incidence was decreased by 50%. This three-pronged strategy was associated with 50 (95% CI: 46, 54) CS cases per 100,000 births and a 73% (95% CI: 71, 75) reduction compared to the base case (Table 2; Figure 2).

#### Four-pronged strategy

The four-pronged strategy that averted the most CS cases consisted of increased prenatal screening coverage, earlier prenatal testing, increased treatment completion, and improved test sensitivity and specificity. Total prenatal screening coverage was increased from 57% to 95%, prenatal screening was performed 75% of the time in the first and second trimester, treatment completion rates were 95%, and test sensitivity and specificity were 99%. This four-pronged strategy was associated with 27 (95% CI: 24, 30) CS cases per 100,000 live births and an 85% reduction (95% CI: 84, 87) compared to the base case. This was the only strategy that met the WHO and NSCP target of less than 30 CS cases per 100,000 live births.

#### Comparison to national syphilis control plan combination strategies

Based on the 2015 and 2020 NSCP strategies laid out by the Chinese Ministry of Health, parameter values were extracted and input into the decision analytic model (Table 2). These parameter changes include incrementally increasing prenatal screening coverage to 86%, incrementally increasing treatment completion rates to 92%, and decreasing incidence rates. The NSCP policies were effective in reducing the number of CS cases to 148 and 105 CS cases per 100,000 live births for the years 2015 and 2020, respectively (Figure 2). Implementing the 2015 policy strategies in the model reduced the CS caseload by 20%, while implementing the 2020 policy strategies reduced the CS caseload by an additional 23%. Figure 2 compares the estimated reduction expected from implementing NSCP strategies with their goals, and illustrates that NSCP strategies alone are insufficient to achieve the desired goals. The four-pronged approach that consisted of increased prenatal syphilis testing, earlier screening, higher treatment completion rate, and increased test specificity and sensitivity was able to achieve the WHO benchmark of <30 CS cases per 100,000 live births.

## Discussion

Our model suggests that prenatal syphilis screening coverage is the single most important component of CS control, but several other strategies, including earlier screening and improved treatment completion, are needed to achieve targets established by the Chinese government. Increasing prenatal screening coverage to 95% resulted in an estimated 78 (95% CI: 73, 83) CS cases per 100,000 live births, compared to the estimated 184 (95% CI: 172, 196) CS cases per 100,000 live births with current screening coverage (57%). Catalyzed by momentum from the NSCP and subsequent increased local resources for syphilis control, China is uniquely positioned to implement far-reaching CS control programs that could have a major impact on maternal and child health [17]. While previous studies have analyzed the relationship between screening coverage and CS cases [10],[18], to our knowledge this is the first study to analyze the relative contribution of individual and comprehensive strategies to overall syphilis control, and the first syphilis control model with empirical validation datasets available, representing a substantial improvement on earlier studies.

Our analysis suggests that improving prenatal syphilis screening is the most important individual component of CS control. This is likely related to suboptimal baseline screening and the critical position of screening in downstream control efforts [19],[20]. Although there have been reports of duplicate reporting of cases and misdiagnosis in China [12], our findings are consistent with net underreporting, with an estimated reporting rate of 75.5%. Some reasons for underreporting include misdiagnosis, lack of CS testing, and non-hospital deliveries. Our model input parameters were conservative and did not account for the underreporting of adult syphilis cases or birth rates [21], and hence CS case outcomes are likely to be an underestimation. While other studies have modeled syphilis screening as part of a comprehensive strategy [4], the relative contribution of screening has not been investigated to date. Achieving 95% screening coverage rates in prenatal settings exceeds the Chinese policy goals of 60% screening coverage in rural areas and 80% screening coverage in urban areas. The 95% screening coverage target is not only necessary but feasible, and has been implemented in similar rural and urban settings. The pilot study in urban Shenzhen City achieved 94% prenatal screening coverage, while another study conducted in rural Guangdong achieved 96% prenatal screening coverage [10],[22]. Globally, greater than 90% prenatal screening coverage rates have been implemented in pilot programs in several low- and middle-income countries [4],[22]. A simple, inexpensive, rapid on-site syphilis test has been rolled out in China and could help realize the universal syphilis screening required at hygiene stations and other low-level prenatal clinics that may not be able to implement non-treponemal syphilis screening [23], but improvement in point-of-care test sensitivity is needed. A United Nations report in 2010 estimated that there is 92% antenatal care coverage across China and suggested the feasibility of expanding syphilis screening coverage across China [24].

Next, earlier syphilis treatment is a critical variable for averting CS burden. Previous models of syphilis control have not explicitly targeted first and second trimester syphilis screening [4],[7],[8]. Many pregnant women in China initiate prenatal care late in pregnancy [25]. Earlier prenatal care would be useful not only for syphilis screening, but also for routine prenatal care [26]–[28]. Treatment in the third trimester is minimally effective in reducing CS transmission to the infant [29]–[32] because much of vertical syphilis transmission occurs prior to the third trimester [33]. At the same time, there are benefits to third trimester screening that include detection of reinfection and prevention of subsequent transmission, in addition to early treatment of infected infants. Early syphilis screening has been proven feasible in resource-constrained settings. A Zambian study successfully implemented earlier prenatal screening through educational campaigns, increasing prenatal syphilis screening before the first 16 wk of gestation from 9.4% to 42.5% [34]. Earlier prenatal screening in Mongolia also resulted in reduced CS cases [35]. Delayed prenatal care is a particular problem among migrant women in China, many of whom have an increased risk of syphilis [36]. The WHO Global Strategy for the Elimination of Congenital Syphilis also calls for early, high-quality prenatal care to increase the impact of syphilis screening [37]. More operational research is needed in order to implement earlier routine prenatal care, especially among migrant women in China.

Treatment completion was also found to reduce CS cases, and previous studies in global settings have demonstrated the potential feasibility of attaining high treatment completion rates in low-income settings [22],[38]–[40]. In the Shenzhen intervention trial, the treatment completion rate was 92% [10]. Increasing treatment completion rates will require the strengthening of local health systems in order to enhance health professional training and identify sustainable financing. Mistrust of syphilis test results, physicians, and clinics has been observed in China, and may contribute to incomplete treatment [41]. In addition, suboptimal test kits were problematic in the past in Guangdong Province. Newly available point-of-care testing provides a higher sensitivity and specificity than many existing test technologies [4].

There are limitations as to how effective small pilot studies can be applied on a larger scale in China and across diverse populations around the world. The presence of existing infrastructure and political will, as well as public acceptance of health outreach programs are critical components for attaining high screening coverage and completion of treatment. Widespread scale-up of successful prenatal syphilis screening pilot programs and clinical services that are trusted among pregnant women within most-at-risk populations are needed in order to overcome these challenges [10]. The NSCP consists of policies targeted at decreasing adult syphilis rates and educational campaigns aimed at increasing test-seeking probabilities. These interventions may decrease adult syphilis cases, but appear to have a limited effect on reducing CS rates, as one-way sensitivity analysis results demonstrated that they avert no more than 7% of the estimated CS case burden, and two-way sensitivity analysis with increased screening coverage demonstrated that they avert no more than 3% of estimated CS case burden. Nonetheless, the additional benefits of reducing the incidence of syphilis among the adult population should not be overlooked—improved sexual health and possibly reduced HIV transmission risk.

The most effective three-pronged strategy fell slightly short of the Chinese Ministry of Health target of <30 cases per 100,000 births, but the four-pronged strategy consisting of increased prenatal syphilis screening coverage, earlier prenatal testing, increased treatment completion rates, and increased test specificity and sensitivity managed to surpass that target, with a model-estimated CS outcome of 27 cases per 100,000 live births. This particular four-pronged strategy was also the most successful of all the combinations: no five-pronged strategy resulted in more CS case aversions. The model suggests that this combination strategy is the strategy that would most likely be associated with achieving the Chinese Ministry of Health goals.

This study has several important limitations. First, as with any model-based analysis, there are inherent uncertainties associated with the structure and necessary simplifying assumptions used for model analysis of a complex problem. In particular, pregnant women and non-pregnant women may have different clinical presentations or test-seeking behaviors. No model can simulate all aspects of real-world interactions. However, simplified models provide a useful tool for understanding the dynamics of relationships between interacting components, which facilitates greater understanding of complex phenomena. Based on current understanding and data from the literature, we included key aspects of syphilis natural history and treatment as well as critical features of intervention strategies, both those laid out in the NSCP and other proven intervention strategies. Confidence intervals for each model estimate were calculated, and the resulting uncertainty in the model did not affect the results. Second, this study generated estimates only for Guangdong Province in China and so may not be applicable to other regions with different syphilis epidemiology, health-seeking behaviors, or other health systems issues. At the same time, our estimates could potentially be applied to similar Chinese urban settings where , as in Guangdong Province, there is a substantial burden of syphilis. Third, our model addresses the impact of syphilis control strategies on the number of CS cases averted, but it does not include other adverse outcomes avoided and all the benefits associated with syphilis control policies in pregnancy. There was a lack of data from Chinese studies that described other adverse outcomes associated with syphilis such as stillbirths and prematurity. Fourth, this study did not collect or evaluate costing data, but other Guangdong research has shown that prenatal syphilis screening is highly cost-effective [8]. Prenatal syphilis testing has also been found to be cost-effective in a variety of settings from Europe to Africa and across a range of syphilis prevalence settings [4],[7],[42]–[45]. Lastly, this study could not quantify the health systems strengthening and improved case management that are also likely to play an important role in syphilis control programs [4]. Health systems strengthening would be useful for CS prevention as well as stemming the syphilis epidemic among pregnant women.

While our analysis specifically focuses on Guangdong Province, these results have broader implications within China and for other middle-income nations. Our analysis illustrates the importance of using multi-pronged approaches to address the complex problem of syphilis control and to reduce the number of CS cases. Reducing the burden of CS would also help achieve child and maternal health goals established by the United Nations [46] and the Asian Development Bank [47].

CS control is a solvable public health problem, but critical details about metrics and screening strategies are required for successful implementation. China's high level of commitment to syphilis control and extensive public health infrastructure create unparalleled opportunities for progress. The targets in the NSCP are consistent with WHO recommendations and represent useful goals. However, our model suggests that achieving these goals may require broadening and further refining of public health strategies for syphilis control.

## Supporting Information

Figure S1 **Sensitivity analysis: varying prenatal screening coverage.** This figure shows the estimated decrease in CS births resulting from increased prenatal screening coverage, which was the single most significant policy intervention.
(TIF)

## Abbreviations

CS: congenital syphilis
NSCP: National Syphilis Prevention and Control Plan
STI: Sexually Transmitted Infection
WHO: World Health Organization

## References

[1] Tucker JD, Chen XS, Peeling RW (2010) Syphilis and social upheaval in china. N Engl J Med 362: 1658–1661. 20445179 10.1056/NEJMp0911149

[2] Temmerman M, Gichangi P, Fonck K, Apers L, Claeys P, et al (2000) Effect of a syphilis control programme on pregnancy outcome in Nairobi, Kenya. Sex Transm Infect 76: 117–121. 10858713 10.1136/sti.76.2.117 PMC1758283

[3] Watson-Jones D, Changalucha J, Gumodoka B, Weiss H, Rusizoka M, et al (2002) Syphilis in pregnancy in tanzania. I. Impact of maternal syphilis on outcome of pregnancy. J Infect Dis 186: 940–947. 12232834 10.1086/342952

[4] Hawkes S, Matin N, Broutet N, Low N (2011) Effectiveness of interventions to improve screening for syphilis in pregnancy: a systematic review and meta-analysis. Lancet Infect Dis 11: 684–691. 21683653 10.1016/S1473-3099(11)70104-9

[5] Tucker JD, Cohen MS (2011) China's syphilis epidemic: epidemiology, proximate determinants of spread, and control responses. Curr Opin Infect Dis 24: 50–55. 21150594 10.1097/QCO.0b013e32834204bf PMC3103765

[6] Kamb ML, Newman LM, Riley PL, Mark J, Hawkes SJ, et al (2010) A road map for the global elimination of congenital syphilis. Obstet Gynecol Int 2010: 312798. 20706693 10.1155/2010/312798 PMC2913802

[7] Rydzak CE, Goldie SJ (2008) Cost-effectiveness of rapid point-of-care prenatal syphilis screening in sub-Saharan Africa. Sex Transm Dis 35: 775–784. 18607319 10.1097/OLQ.0b013e318176196d

[8] Hong FC, Liu JB, Feng TJ, Liu XL, Pan P, et al (2010) Congenital syphilis: an economic evaluation of a prevention program in China. Sex Transm Dis 37: 26–31. 19734825 10.1097/OLQ.0b013e3181b3915b

[9] Weinstein MC, O'Brien B, Hornberger J, Jackson J, Johannesson M, et al (2003) Principles of good practice for decision analytic modeling in health-care evaluation: report of the ISPOR task force on good research practices—modeling studies. Value Health 6: 9–17. 12535234 10.1046/j.1524-4733.2003.00234.x

[10] Cheng JQ, Zhou H, Hong FC, Zhang D, Zhang YJ, et al (2007) Syphilis screening and intervention in 500,000 pregnant women in Shenzhen, the People's Republic of China. Sex Transm Infect 83: 347–350. 17693449 10.1136/sti.2006.023655 PMC2659022

[11] Yang LG, Tucker JD, Yang B, Shen SY, Sun XF, et al (2010) Primary syphilis cases in Guangdong Province 1995–2008: opportunities for linking syphilis control and regional development. BMC Public Health 10: 793. 21192782 10.1186/1471-2458-10-793 PMC3022862

[12] Wu ZY, Zhou PY (2010) Syphilis and social upheaval in china. N Engl J Med 363: 1088–1089. 10.1056/NEJMc1006525 20825326

[13] Wu DD, Hong FC, Feng TJ, Liu XL, Lin LJ, et al (2010) Congenital syphilis: refining newborn evaluation and management in Shenzhen, southern China. Sex Transm Infect 86: 280–284. 20576913 10.1136/sti.2009.038893

[14] Hong FC, Feng TJ, Cai YM, Wen LZ, Pan P, et al (2009) Burden of syphilis infections in Shenzhen, China: a preliminary estimation. Int J STD AIDS 20: 115–118. 19182058 10.1258/ijsa.2008.008252

[15] Larsen SA, Steiner BM, Rudolph AH (1995) Laboratory diagnosis and interpretation of tests for syphilis. Clin Microbiol Rev 8: 1–21. 7704889 10.1128/cmr.8.1.1 PMC172846

[16] Chapel TA (1980) The signs and symptoms of secondary syphilis. Sex Transm Dis 7: 161–164. 7455863 10.1097/00007435-198010000-00002

[17] Chen ZQ, Zhang GC, Gong XD, Lin C, Gao X, et al (2007) Syphilis in China: results of a national surveillance programme. Lancet 369: 132–138. 17223476 10.1016/S0140-6736(07)60074-9 PMC7138057

[18] Deperthes BD, Meheus A, O'Reilly K, Broutet N (2004) Maternal and congenital syphilis programmes: case studies in Bolivia, Kenya and South Africa. Bull World Health Organ 82: 410–416. 15356932 PMC2622863

[19] US Centers for Disease Control and Prevention (2010) 2010 STD treatment guidelines. Atlanta: US Centers for Disease Control and Prevention.

[20] World Health Organization (2010) Elimination of congenital syphilis. Geneva: World Health Organization.

[21] Lin CC, Gao X, Chen XS, Chen Q, Cohen MS (2006) China's syphilis epidemic: a systematic review of seroprevalence studies. Sex Transm Dis 33: 726–736. 16755273 10.1097/01.olq.0000222703.12018.58

[22] Mabey DC, Sollis KA, Kelly HA, Benzaken AS, Bitarakwate E, et al (2012) Point-of-care tests to strengthen health systems and save newborn lives: the case of syphilis. PLoS Med 9: e1001233 doi:10.1371/journal.pmed.1001233. 22719229 10.1371/journal.pmed.1001233 PMC3373627

[23] Tucker JD, Hawkes SJ, Yin YP, Peeling RW, Cohen MS, et al (2010) Scaling up syphilis testing in China: implementation beyond the clinic. Bull World Health Organ 88: 452–457. 20539859 10.2471/BLT.09.070326 PMC2878148

[24] United Nations Statistics Division (2010) Antenatal care coverage (ANC) [database]. Available: http://data.un.org/Data.aspx?d=SOWC&f=inID%3A77. Accessed 1 July 2012.

[25] Cui Y, Yang L, Lu MT (2009) [Analysis on the status of antenatal checkup program in certain areas of China, 2005]. Zhonghua Liu Xing Bing Xue Za Zhi 30: 887–890. 20193220

[26] Li CG, Li CF, Li Q, Li M (2009) Thalassemia incidence and treatment in China with special reference to Shenzhen City and Guangdong province. Hemoglobin 33: 296–303. 19814675 10.3109/03630260903211698

[27] Yanping W, Lei M, Li D, Chunhua H, Xiaohong L, et al (2010) A study on rural-urban differences in neonatal mortality rate in china, 1996–2006. J Epidemiol Community Health 64: 935–936. 20584731 10.1136/jech.2009.093138

[28] Tang J, Li NX (2008) [Use of maternal health care services in poor regions in Sichuan.]. Sichuan Da Xue Xue Bao Yi Xue Ban 39: 1004–1006. 19253847

[29] Wendel GD Jr, Sheffield JS, Hollier LM, Hill JB, Ramsey PS, et al (2002) Treatment of syphilis in pregnancy and prevention of congenital syphilis. Clin Infect Dis 35 (Suppl 2) S200–S209. 12353207 10.1086/342108

[30] Liu JB, Hong FC, Pan P, Zhou H, Yang F, et al (2010) A risk model for congenital syphilis in infants born to mothers with syphilis treated in gestation: a prospective cohort study. Sex Transm Infect 86: 292–296. 20460262 10.1136/sti.2009.037549

[31] Alexander JM, Sheffield JS, Sanchez PJ, Mayfield J, Wendel GD Jr (1999) Efficacy of treatment for syphilis in pregnancy. Obstet Gynecol 93: 5–8. 9916946 10.1016/s0029-7844(98)00338-x

[32] Sheffield JS, Sanchez PJ, Morris G, Maberry M, Zeray F, et al (2002) Congenital syphilis after maternal treatment for syphilis during pregnancy. Am J Obstet Gynecol 186: 569–573. 11904625 10.1067/mob.2002.121541

[33] Walker DG, Walker GJ (2002) Forgotten but not gone: the continuing scourge of congenital syphilis. Lancet Infect Dis 2: 432–436. 12127355 10.1016/s1473-3099(02)00319-5

[34] Hira SK, Bhat GJ, Chikamata DM, Nkowane B, Tembo G, et al (1990) Syphilis intervention in pregnancy: Zambian demonstration project. Genitourin Med 66: 159–164. 2370060 10.1136/sti.66.3.159 PMC1194495

[35] Munkhuu B, Liabsuetrakul T, Chongsuvivatwong V, McNeil E, Janchiv R (2009) One-stop service for antenatal syphilis screening and prevention of congenital syphilis in Ulaanbaatar, Mongolia: a cluster randomized trial. Sex Transm Dis 36: 714–720. 19773681 10.1097/OLQ.0b013e3181bc0960

[36] Zhu L, Qin M, Du L, Xie RH, Wong T, et al (2010) Maternal and congenital syphilis in Shanghai, China, 2002 to 2006. Int J Infect Dis 14 (Suppl 3) e45–e48. 20137991 10.1016/j.ijid.2009.09.009

[37] World Health Organization (2007) The global elimination of congenital syphilis: rationale and strategy for action. Geneva: World Health Organization.

[38] Wilkinson D, Sach M (1998) Improved treatment of syphilis among pregnant women through on-site testing: an intervention study in rural South Africa. Trans R Soc Trop Med Hyg 92: 348. 9861415 10.1016/s0035-9203(98)91038-0

[39] Fitzgerald DW, Behets F, Preval J, Schulwolf L, Bommi V, et al (2003) Decreased congenital syphilis incidence in Haiti's rural Artibonite region following decentralized prenatal screening. Am J Public Health 93: 444–446. 12604493 10.2105/ajph.93.3.444 PMC1447761

[40] Bronzan RN, Mwesigwa-Kayongo DC, Narkunas D, Schmid GP, Neilsen GA, et al (2007) On-site rapid antenatal syphilis screening with an immunochromatographic strip improves case detection and treatment in rural South African clinics. Sex Transm Dis 34 (Suppl 7) S55–S60. 17139234 10.1097/01.olq.0000245987.78067.0c

[41] Tucker JD, Yang LG, Yang B, Young D, Henderson GE, et al (2011) Prior HIV testing among STD patients in Guangdong Province, China: opportunities for expanding detection of sexually transmitted HIV infection. Sex Transm Dis 39: 182–187. 10.1097/OLQ.0b013e318237b3b4 PMC3282013 22337103

[42] Schmid G (2004) Economic and programmatic aspects of congenital syphilis prevention. Bull World Health Organ 82: 402–409. 15356931 PMC2622861

[43] Blandford JM, Gift TL, Vasaikar S, Mwesigwa-Kayongo D, Dlali P, et al (2007) Cost-effectiveness of on-site antenatal screening to prevent congenital syphilis in rural eastern Cape Province, Republic of South Africa. Sex Transm Dis 34 (Suppl 7) S61–S66. 17308502 10.1097/01.olq.0000258314.20752.5f

[44] Schackman BR, Neukermans CP, Fontain SN, Nolte C, Joseph P, et al (2007) Cost-effectiveness of rapid syphilis screening in prenatal HIV testing programs in Haiti. PLoS Med 4: e183 doi:10.1371/journal.pmed.0040183. 17535105 10.1371/journal.pmed.0040183 PMC1880854

[45] Vickerman P, Peeling RW, Terris-Prestholt F, Changalucha J, Mabey D, et al (2006) Modelling the cost-effectiveness of introducing rapid syphilis tests into an antenatal syphilis screening programme in Mwanza, Tanzania. Sex Transm Infect 82 (Suppl 5)v38–v43. 17215276 10.1136/sti.2006.021824 PMC2563909

[46] United Nations (2011) What they are [Millennium Development Goals]. Available: http://www.unmillenniumproject.org/goals/index.htm. Accessed 1 July 2012.

[47] Asian Development Bank (2012) Health. Available: http://www.adb.org/sectors/health/main. Accessed 1 July 2012.

[48] Parish WL, Laumann EO, Cohen MS, Pan S, Zheng H, et al (2003) Population-based study of chlamydial infection in China: a hidden epidemic. JAMA 289: 1265–1273. 12633188 10.1001/jama.289.10.1265

[49] Zhao F, Guo SF, Li BH, Cui Y, Wu KS (2005) [Survey on the situation of antenatal care in different regions of China in 1971–2003]. Zhonghua Liu Xing Bing Xue Za Zhi 26: 172–176. 15941499

[50] Jiao T, Xu X, Han CY, Liu L, Zhou Y, et al (2010) [Perinatal outcomes of pregnant women with syphilis treated by procaine penicillin at different gestations]. Chin J Derm Venereol 24: 934.

[51] Zhang XM, Zhang RN, Lin SQ, Chen SX, Zheng LY (2004) [Clinical analysis of 192 pregnant women infected by syphilis.]. Zhonghua Fu Chan Ke Za Zhi 39: 682–686. 16144566

[52] Li L, Liu M, Wang F (2009) [Clinical analysis on 121 pregnant women infected by syphilis.]. Matern Child Health Care China 24: 4087.

[53] China National STD Control Center (2010) [2010 national STD control and prevention national treatment guidelines]. Nanjing: China National STD Control Center.

