# Supplementary figures and images for: Prioritizing Congenital Syphilis Control in South China: A Decision Analytic Model to Inform Policy Implementation
Source: PLoS Med. 2013 Jan 22;10(1):e1001375. doi: 10.1371/journal.pmed.1001375 (PMC3551934; doi:10.1371/journal.pmed.1001375)

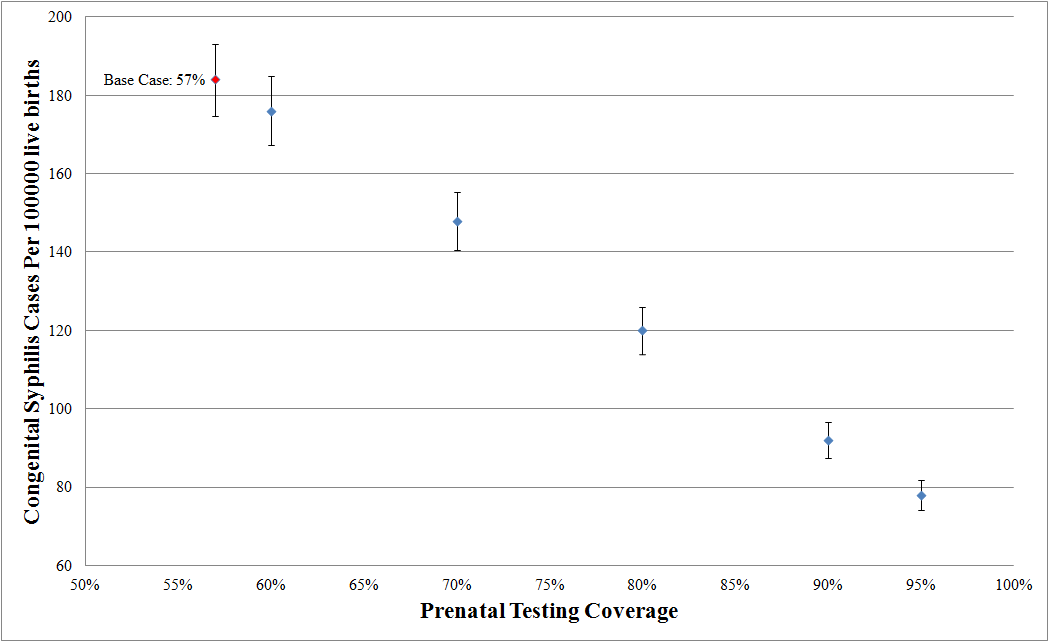

Supplement: Figure S1 — Sensitivity analysis: varying prenatal screening coverage. This figure shows the estimated decrease in CS births resulting from increased prenatal screening coverage, which was the single most significant policy intervention. (TIF) [file pmed.1001375.s001.tif]
